# A Transparent and Transferable Framework for Tracking Quality Information in Large Datasets

**DOI:** 10.1371/journal.pone.0112249

**Published:** 2014-11-07

**Authors:** Derek E. Smith, Stefan Metzger, Jeffrey R. Taylor

**Affiliations:** 1 National Ecological Observatory Network, Boulder, Colorado, United States of America; 2 Institute of Arctic and Alpine Research, University of Colorado, Boulder, Colorado, United States of America; UGent/VIB, Belgium

## Abstract

The ability to evaluate the validity of data is essential to any investigation, and manual “eyes on” assessments of data quality have dominated in the past. Yet, as the size of collected data continues to increase, so does the effort required to assess their quality. This challenge is of particular concern for networks that automate their data collection, and has resulted in the automation of many quality assurance and quality control analyses. Unfortunately, the interpretation of the resulting data quality flags can become quite challenging with large data sets. We have developed a framework to summarize data quality information and facilitate interpretation by the user. Our framework consists of first compiling data quality information and then presenting it through 2 separate mechanisms; a quality report and a quality summary. The quality report presents the results of specific quality analyses as they relate to individual observations, while the quality summary takes a spatial or temporal aggregate of each quality analysis and provides a summary of the results. Included in the quality summary is a final quality flag, which further condenses data quality information to assess whether a data product is valid or not. This framework has the added flexibility to allow “eyes on” information on data quality to be incorporated for many data types. Furthermore, this framework can aid problem tracking and resolution, should sensor or system malfunctions arise.

## Introduction

Advancements in sensor measurement techniques and data collection continue to increase both the accuracy and quantity of measurements we are able to capture. Yet, without some indication of a data set's validity it is seemingly useless. Similarly, while the demand on the quality of data can vary from one research question to another, it is paramount that the data quality is known. One organization that is confronted by this challenge is the National Ecological Observatory Network (NEON). Set to begin full operations in 2017, the NEON is designed to collect and provide a wide array of environmental data freely to the public for a period of 30 years, from sites located throughout the United States [1], [2]. When the observatory is complete, the NEON will consist of over 14,000 automated sensors and stream more than 55,000 variables. The sheer number of measurements that the NEON will capture has in turn necessitated the automation of much of the data processing. While historically data quality has been assessed manually using an “eyes on” approach, this is no longer feasible for datasets of this magnitude. This has subsequently led the NEON to develop various automated quality assessment and quality control (QA/QC) analyses [3]. In turn this has necessitated the development of a framework that automates the propagation and interpretation of data quality information, which is discussed here.

Ideally, in order to evaluate whether data is acceptable, the intended data use should first be identified [4]. Herein lies a major challenge for the NEON because it is impossible to account for all potential ways the resulting data products may be used. Thus, in order to accommodate varying use cases and levels of expertise, we developed a framework that condenses data quality information, while retaining several levels of detail. The reliable generation of data products can be summarized into 3 main components: (i) Quality requirements for the raw data, components/assemblies and data products. (ii) Quality verification of raw data and the final data products, and (iii) Corrective actions taken for data products that do not meet their requirements [5]. Here components (ii) and (iii) are addressed. The data quality framework summarizes quality information by condensing individual sensor tests and QA/QC analyses into quality metrics and generates an overall quality summary, which will be discussed in the Sect. “*Materials and Methods*”. In turn one's ability to assess the overall quality of a data product is vastly simplified through the data quality framework.

NEON has implemented this framework for atmospheric sensor data in order to provide transparent data quality information to its users. Here we present how this framework can be used to assess the data quality of a NEON tower sensor. To put the ability of the framework to condense data quality information into perspective, at a typical NEON site over 150 atmospheric and terrestrial sensor measurements are made. Generally, 1- and 30-minute averages are produced from sensor measurements, which are often acquired at a rate of 1 Hz and include 8 different QA/QC analyses. Accordingly, the QA/QC results for atmospheric and terrestrial sensors are upwards of 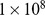 a day, 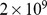 a month, and 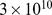 a year. Through the presented framework, the QA/QC information can be condensed by roughly 4 orders of magnitude for 1-minute averages and 6 orders of magnitude for 30-minute averages. It becomes increasingly apparent that one would be unable to digest and interpret all of the individual quality information from sensors sampling at a rate of 20 Hz. Thus, we sought to formulate an automated framework that allows the results from sensor tests and QA/QC analyses to be summarized in a way that is transparent and easily interpretable. This will enable users to determine whether corrective actions are necessary if a data product has not met the requirements needed for a specific use case.

Data quality assessment techniques employed by the eddy-covariance community were used as a foundation for this framework, since they routinely assess large data sets and scrutinize their data quality [6], [7], and [8]. These approaches were designed to determine the quality of a reported data product that originated from multiple input data products. Briefly, these schemes define a set of criteria that individually map the quality of inputs to a data product. This information is then incorporated into a rank-based scheme that determines the overall quality of the reported data product. A caveat of these rank-based approaches is that they do not easily transfer and integrate among different types of measurements, as they are typically designed to be used for 1 specific application.

Therefore, we chose to (initially) refrain from grouping/ranking data into different categories based on the magnitude of their deviation for a specific analysis. Instead, we first derive a simplistic approach that allows the framework to be applied across a greater number of data types. This, in turn, allows for data quality information to be propagated in a less ambiguous manor across varying use cases. The methodology was initially conceived for use with ecological data, nonetheless it can be readily applied to various data types. Even in the scope of NEON, ecological data may not be big data when viewed through the lens of data mining, but it is comparatively complex. As such, it quickly becomes problematic to use existing rank-based approaches to create and implement a standardized and transparent methodology to summarize quality across varying data types. In addition, existing rank-based approaches limit the degree to which quality information can be propagated since the approaches generally require that they are tailored to specific questions, methods, use cases, and so forth.

Here, we advance existing rank-based approaches to create a data quality assessment scheme that is modular in form so it can easily be transferred among a variety of sensor measurements and physical samples. It is also shown how, at a later stage, means for gradation can be re-inserted. For example, the framework can assess and propagate information for sensor measurements and physical samples from an array of sources, e.g., atmospheric, terrestrial, and/or aquatic. In addition, the data quality framework enables all the quality information to be condensed into a final flag. This final quality flag signifies whether a data product is valid or invalid based on predetermined and user-defined thresholds for the various sensor tests and QA/QC analyses that are in place. Here we present the principal components of this framework as well as an example of how it can be used to assess data quality. The majority of the paper will focus on the Sect. “*Materials and Methods*”, which provides an overview of the data quality framework methodology, its various components, and how it is constructed. The Sect. “*Application of Data Quality Framework*” provides an example of how the framework can be applied to ecological data using sensor-based measurements from a NEON tower site. We conclude with discussing the applicability and expandability of the framework to various data types and use cases.

## Materials and Methods

Each of the following sub-sections provide an overview of an individual component that collectively compose the entire data quality framework. As presented, each component of the framework is sequential in nature, incorporating the components from previous sections in order to construct the framework. Initially, quality flags and their various states are defined. This is followed by an overview of how quality flags relate to quality metrics. Quality metric information is then further condensed into a final quality flag for a data product. Lastly, a system for summarizing and presenting the data quality information is presented.

### Quality Flags

Quality flags are defined as quality information provided through sensor tests and/or QA/QC analyses. In Eq. (1) we depict a quality flag for an arbitrary analysis, 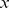, as having 1 of 3 possible states. However, a quality flag with greater or fewer number of states could be incorporated into the framework in the same fashion, an example of this is shown in Sect. “*Application of Data Quality Framework*”.
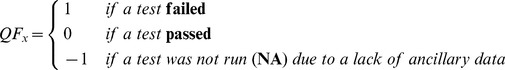
(1)


It is important to note that results from sensor tests and QA/QC analyses can be continuous. In order to discretize such results for use in the present framework, a user-defined threshold or logic for a range of states needs to be applied, such as described in Sect. “*Computation of Final Quality Flag*” and Table 1, respectively.

**Table 1:** Treatment of temperature sensor data based on results from pair-wise comparisons, i.e., Eq. (9)–(11).

Inputs A B C	Averaging Operator	Averaging Flag (*QF* 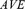 )
T T T	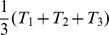	0
T T F	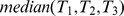	1
T F T	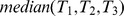	2
T F F	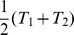	3
F T T	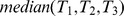	4
F T F	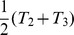	5
F F T	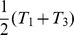	6
F F F	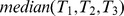	7

### Quality Metrics

Quality metrics simply summarize the quality flag information for a given period of time or spatial extent by their frequency of occurrence. Respectively, there is 1 quality metric for each possible state a quality flag could take, which is represented as a percentage of the total number of measurements. Accordingly, our example of a quality flag in Eq. (1) has 3 corresponding quality metrics (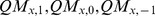), which relate to the individual quality flag's state.
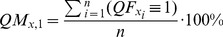
(2)




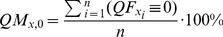
(3)




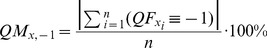
(4)


Where, 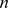 is the number of measurements for a given period of time, and 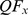 is the quality flag result for a specific quality analysis that corresponds to an individual measurement obtained during the period of time. The total sum for all of the quality metrics related to a specific quality flag is always 100%, independent of the number of states that the quality flags and associated quality metrics may have. The relationship between quality metrics and quality flags for an arbitrary data product with a set of 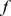 quality flags and 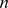 measurements is displayed in Table 2.

**Table 2:** Illustration of how quality flags and quality metrics are determined for a data product with *n* measurements and *f* quality flags.

*Observation*	*QF*_1_			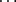	*QF_f_*		
1	1∨0∨−1			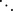	1∨0∨−1		
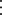	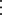			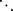	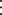		
n	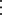			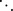	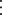		
	*QM*_1,1_	*QM*_1,0_	*QM*_1,−1_		*QM_f_*_,1_	*QM_f_*_,0_	*QM_f_*_,−1_
	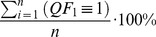	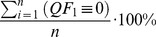	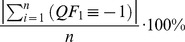	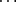	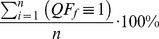	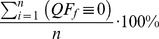	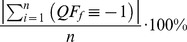

### Alpha and Beta Quality Flags and Metrics

The calculation of 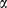 and 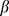 quality flags and quality metrics are very similar to one another and are specific to a measurement. The difference between the 2 of them is that, 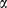 determines whether or not 1 or more of the quality flags failed for a measurement, while 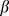 determines whether or not 1 or more of the results of the quality flags could not be determined due to a lack of ancillary data. The calculation of 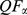 and 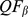 for an individual measurement are shown in Eq. (5) and (6).
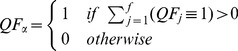
(5)




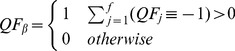
(6)


Following the calculation of 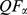 and 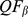, i.e., Eq. (5) and (6), 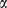 and 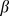 quality metrics, 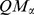 and 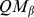, are determined. It is important to mention that for a given observation, 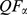 and 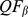 can both be set to 1 in the event an associated quality flag is set to 1 and another associated quality flag could not be computed due to a lack of ancillary data. Thus, while the quality metrics derived from individual quality flags always sum to 100%, quality metrics for 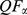 and 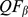 always sum to 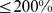. A visual representation of how 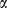 and 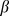 quality flags and quality metrics are determined and fit into the overall framework is shown in Table 3.

**Table 3:** Overview of the data quality framework and how the various sections/modules fit in.

*Observation*	*QF*_1_			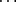	*QF_f_*			*QF_α_*	*QF_β_*
1	1∨0∨−1			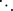	1∨0∨−1			*Eq*. (5)	*Eq*. (6)
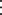	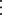			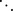	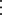			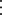	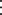
n	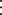			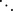	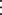			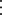	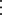
	*Eq*. (2)	*Eq*. (3)	*Eq*. (4)	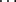	*Eq*. (2)	*Eq*. (3)	*Eq*. (4)	*Eq*. (2)	*Eq*. (2)

### Computation of Final Quality Flag

To increase the comprehensiveness of the data quality framework, information is then further condensed into a final quality flag. The final quality flag, 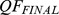, offers users a way to quickly assess the quality of a data product and to determine whether or not data has passed or failed an overall assessment. The 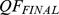 threshold is defined by relating 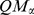 and 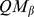 in a meaningful way to the variable of interest. Therefore, the validity of a data product is based on an acceptable amount of failed quality tests, and subsequently removed observations, as well as an acceptable amount of inconclusive tests. If a data product reaches or exceeds this threshold it is flagged as invalid (i.e., 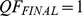) and valid (i.e., 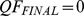) otherwise. The threshold is user-defined, which enables its application across a multitude of varying data types.

**Figure 1:**
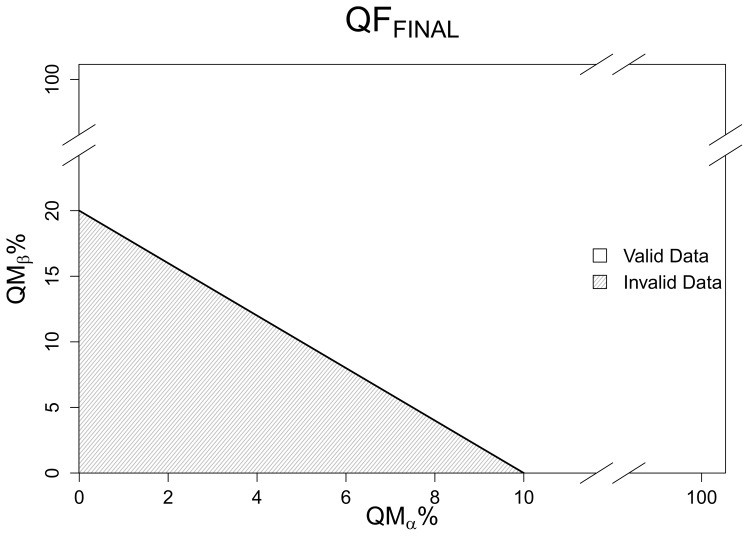
Representation of 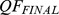 using Eq. (7) to determine whether or not a data product is valid.

Here we present the 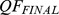 threshold arbitrarily as a 2∶1 ratio of 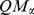 to 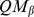 with maximum fractions of 10% 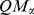 and 20% for 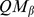, Eq. (7). Hence, in this example a data product is considered invalid if 10% or more of the data used to create it has failed a set of quality tests and were removed, more than 20% of quality test results remain inconclusive, or a combination of the 2. Figure 1 represents the ratio of 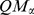 to 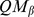 in a graphical form for this threshold.
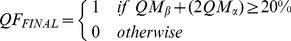
(7)


### Quality Summary and Quality Report

Ultimately, all of the quality information for a data product (i.e., quality flags and quality metrics) and the final quality flag, are compiled and summarized in 2 separate schemes; a quality report and a quality summary. The quality report presents the result of all sensor tests and QA/QC analyses as they relate to individual observations, i.e., the individual quality flag results. For example, a quality report for a 30-minute temperature average, sampled at a rate of 1 Hz, allows users to differentiate the 1800 outcomes for each sensor test and QA/QC analysis. Alternatively, the quality summary provides the quality metric results for each quality flag as well as the final quality flag, which allows for a quick assessment of the validity of a data product. An overview of what information is contained in the quality report and quality summary is shown in Table 4. This approach presents and retains several levels of detail, which facilitates data transparency and usability. In addition, this allows backtracking through the results, thus aiding problem tracking and resolution, as shown in the Sect. “*Application of Data Quality Framework*”.

**Table 4:** Overview of the information contained in the quality summary and quality report.

*Observation*	*QF*_1_			*QF*_2_			*QF*_3_			*QF_α_*	*QF_β_*	
1	0			0			0			0	0	
2	0			0			0			0	0	
3	0			0			0			0	0	
6	0			0			0			0	0	
7	0			0			0			0	0	
8	0			0			1			1	0	
9	1			0			1			1	0	
10	1			0			0			1	0	
11	0			0			0			0	0	
12	0			0			0			0	0	
13	0			0			0			0	0	
14	0			0			0			0	0	
15	0			−1			−1			0	1	
	*QM*_1,1_ 13%	*QM*_1,0_ 87%	*QM*_1,−1_ 0%	*QM*_2,1_ 0%	*QM*_2,0_ 93%	*QM*_2,−1_ 7%	*QM*_3,1_ 13%	*QM*_3,0_ 80%	*QM*_3,−1_ 7%	*QM* *_α_* 20%	*QM* *_β_* 7%	*QF_FINAL_* 1

This example displays the quality report and quality summary information for 15 sensor measurements and 3 arbitrary quality analyses. The quality report contains the individual quality flag outcomes for each sensor measurement, i.e., rows 1–15. The quality summary includes the corresponding quality metrics and the final quality flag information, i.e., the bottom row.

**Figure 2:**
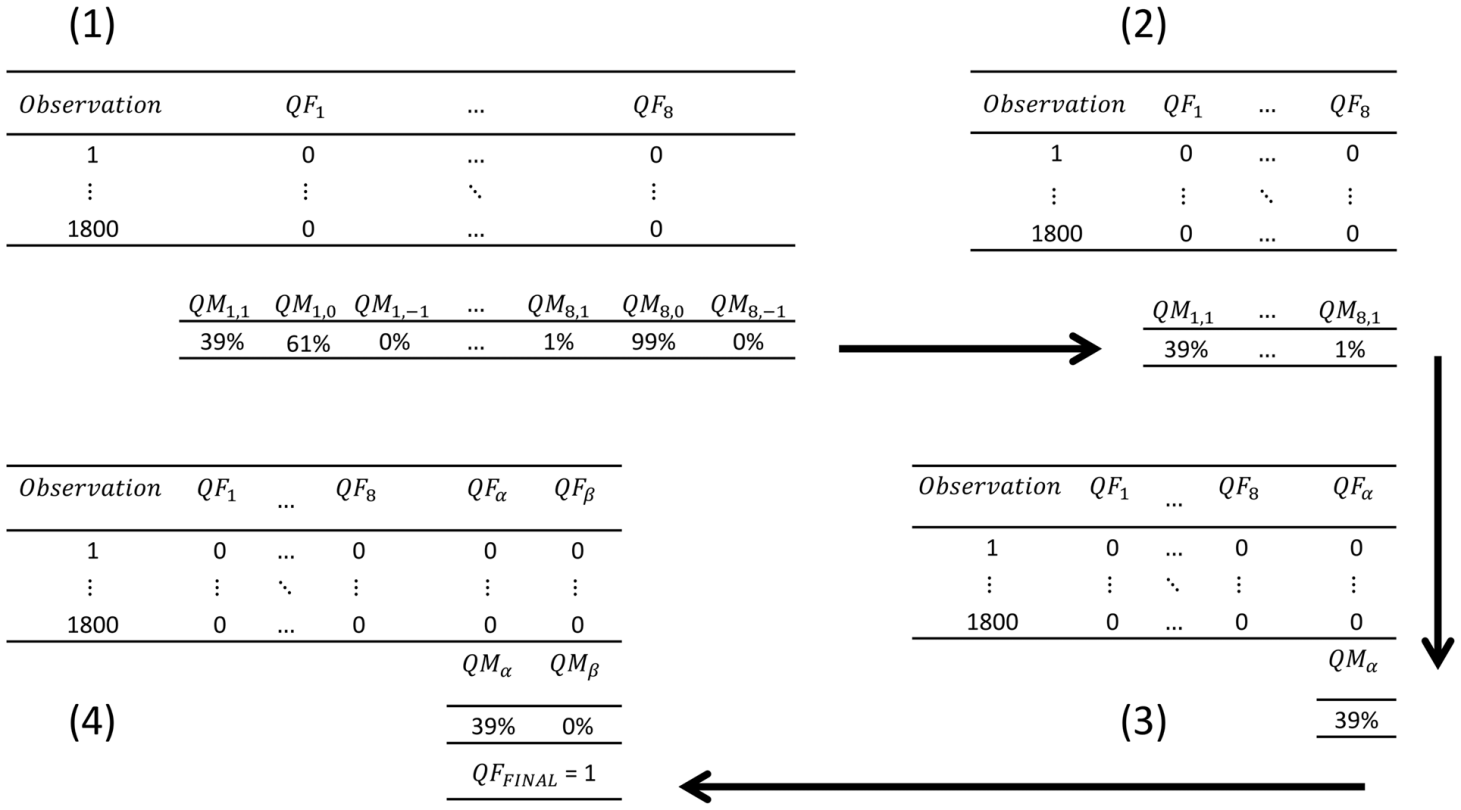
Different ways data quality information can be condensed for a 30-minute average of a sensor sampled at 1 Hz with 8 quality flags. (1) The 14400 flag outcomes are condensed into 24 quality metrics. (2) The 14400 flag outcomes are condensed down to 8 quality metrics representing failed analyses only. (3) The 14400 flag outcomes are reduced to 1 quality metric representing the percentage of sensor measurements that failed 1 or more analysis. (4) The 14400 flag outcomes are condensed to a final quality flag.

Furthermore, depending on the use case the amount of quality information reported can be substantially reduced by including only specific information. For example, a sensor sampled at 1 Hz reports 1800 measurements for a 30-minute average. If 1 sensor test and 7 different QA/QC analyses are run on the individual observations, a 30-minute average has 14400 related quality flag outcomes. Applying the data quality framework, the 14400 outcomes are summarized into 24 quality metrics (Figure 2, panel 1). Similarly, if one is only interested in sensor measurements that failed sensor tests and QA/QC analyses, the number of quality metrics can be reduced to 8, i.e., the 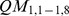 (Figure 2, panel 2). The outcomes of the quality flags can be further reduced to a single quality metric if one is only interested in the percent of sensor measurements that have 1 or more failed quality flags, (Figure 2, panel 3). Additionally, the data quality information can be entirely condensed into a single quality flag by applying the final quality flag concept (Figure 2, panel 4).

### Data Description

Preliminary, error-injected data from a NEON tower site in Sterling, Colorado, from January 2013, is used to illustrate how this framework can be applied to ecological data (Data S1). The Sterling tower site (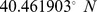, 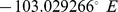, altitude: 1364 m asl) is located in an agricultural field in the eastern plains of Colorado. Here, the triple redundant aspirated air temperature (TRAAT) measurement, which is located on the top of the Sterling tower, is assessed. The temperature measurement is captured at a rate of 1 Hz, however the techniques presented here can be applied to any sampling rate.

The tower top temperature measurement is made via 3 platinum resistance thermometers (PRTs) that are housed in a fan aspirated radiation shield. Under the NEON's current framework each 1 Hz TRAAT observation has 1 sensor test and 8 associated QA/QC analyses, which inform the overall data quality of 1- and 30-minute average data products. However, for simplicity here we only examine the outcome of 3 of these quality analyses for a 10-minute TRAAT average. First, the range test flags TRAAT measurements that are outside of a realistic temperature range for a given location and season. Second, the null test assesses the number of dropped observations over a given period of time. Third, the averaging flag indicates how the final 1 Hz TRAAT measurement is calculated from the 3 PRT measurements. The averaging flag consists of 8 possible outcomes, which represent the different ways the PRTs can be averaged to determine a 1 Hz TRAAT measurement. In order to understand how the 8 averaging flag outcomes originate, the algorithm used to calculate a TRAAT measurement is briefly discussed.

Following the conversion of the 3 PRT measurements to degrees Celsius, pairwise differences are computed among the PRT measurements (i.e., 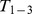) with corresponding time stamps, as follows:
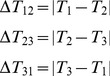
(8)


Next, the differences among the sensor measurements are compared to their associated uncertainties (i.e., 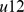, 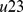 and 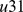), and true and false statements are used to determine whether the PRTs within the TRAAT sensor are operating normally. Under normal operation, if sufficient aspiration is present and the PRTs are functioning properly, the variation among the 3 sensor measurements should not be greater than their associated measurement uncertainties, Eq. (9)–(11).

The uncertainty of the PRT measurements can be attributed to a number of quantifiable and unquantifiable sources. For example, we can quantify uncertainties that arise from the sensor calibration and the data acquisition system. Alternatively, temperature bias from radiative forcing can be minimized through active aspiration. Yet, we cannot entirely quantify its residual effect on the sensor measurement. NEON is currently in the process of determining uncertainty estimates for both PRT calibrations as well as the data acquisition system. Thus, for this example, a manufacturer accuracy specification of 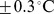 at 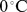 in addition to an estimated aspiration bias of 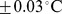 are combined to an uncertainty threshold of 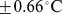 for the pairwise differences. However, once the sensor calibration and data acquisition system uncertainty have been fully characterized, the uncertainty estimates will become specific to each individual sensor and temperature specific.
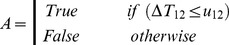
(9)




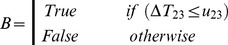
(10)




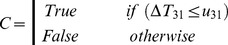
(11)


Once the differences among sensor measurements have been compared to their respective uncertainties, the results from Eq. (9)–(11) are evaluated against the logic shown in Table 1. This determines which averaging operator is used to compute the resulting, 1 Hz averaged TRAAT measurement. For problem tracking purposes an averaging flag is set to correspond to the averaging operator that was used, which under normal operation should remain set to zero.

**Figure 3:**
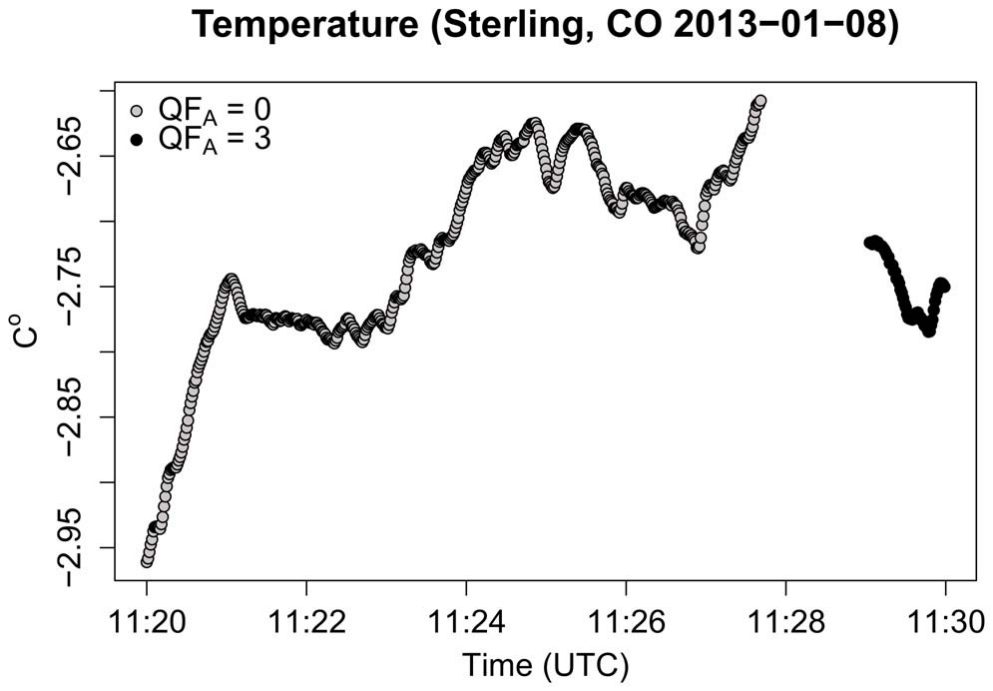
TRAAT from the NEON Sterling CO site, each circle indicates an averaged 1 Hz sensor measurement. The open gray circles indicate that the average of the 3 PRTs was used in the TRAAT calculation. Alternatively, the full black circles indicate that the TRAAT 1 Hz sensor measurement was calculated as the average of PRT 1 and PRT 2, i.e., averaging flag set to 3 in Table 1.

The assessed 10-minute period of error-injected TRAAT data consists of 600 1 Hz temperature averages and 1800 individual PRT sensor measurements (Figure 3). Since quality analyses are applied to the averaged 1 Hz TRAAT measurements, a total of 1800 quality flag outcomes associated with the averaging flag (600), range (600), and null (600) quality analyses were generated for the 10-minute temperature average of 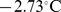.

### Application of Data Quality Framework

Applying the quality framework to the TRAAT sensor tests and QA/QC analyses, the 1800 quality flag outcomes were condensed to the 14 quality metrics shown in Table 5. 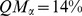 and 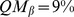 were determined based on the criterion shown in Eq. (12) and (13). Using Eq. (7) we found 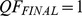, i.e., the 10-minute TRAAT average was deemed not valid.
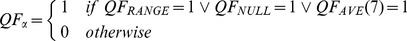
(12)


**Table 5:** The associated quality metrics for the 10-minute TRAAT average.

Quality Metric	Outcome (%)
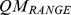 (0)	100
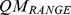 (1)	0
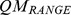 (−1)	0
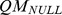 (0)	86
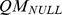 (1)	14
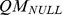 (−1)	0
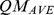 (0)	77
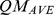 (1)	0
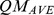 (2)	0
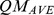 (3)	9
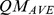 (4)	0
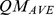 (5)	0
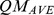 (6)	0
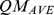 (7)	0

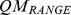
 represent the quality metric results from the range test, 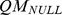 represent the quality metric results from the null test, and 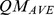 represent the quality metric results from the averaging flag as shown in Table 1.



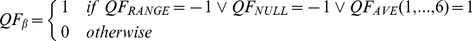
(13)


The quality metrics indicated that the observed PRT temperatures remained within a realistic range throughout 10-minute time period. However, the quality metric for the null analysis indicated that 14% of the observations were missing. In order to determine when the null flags occurred the quality report was examined to identify the individual flag results. The quality report indicated that all of the null flags were consecutive, which was verified when plotting the averaged 1 Hz TRAAT measurements (Figure 3).

Assessing the quality metrics produced by the averaging flag indicated that the majority of 1 Hz TRAAT measurements were made by averaging all 3 PRTs. However, 9% of the observations had an averaging flag of 3, indicating that only PRT 1 and PRT 2 within the TRAAT sensor were used (Table 5). The averaging flag was used to backtrack which of the 1 Hz TRAAT averages were calculated from less than 3 PRT observations (highlighted in black in Figure 3).

Through the assessment of the quality analyses it became apparent that after a series of consecutive nulls, the averaged TRAAT measurements changed from being determined by the average of the 3 PRTs to the average of only 2 of the PRTs. This may indicate that either a PRT or another part of the sensor malfunctioned. In practice, data following this 10-minute average should be inspected to determine whether it is necessary to issue a trouble ticket and have the TRAAT sensor physically inspected.

The error-injected result from the TRAAT sensor is just 1 example of the quality framework's ability to allow a user to more efficiently assess data quality. If the final quality flag indicates that there may be issues with the data, one can subsequently inspect the quality summary and quality report to extract specific information. This enables one to efficiently identify areas of interest for detailed inspection, instead of having to inspect the entire data set. In addition, as seen with the TRAAT results, the quality framework can expedite trouble shooting and backtracking procedures, as well as assist with algorithm development. For example, if one wanted to test new QA/QC analyses the quality summary could aid in quickly identifying whether or not these analyses were generating too many false positives and/or negatives, while the quality report could be used to target the specific problem areas.

## Discussion and Conclusions

Coupled with the increasing number of measurements collected by researchers, comes the need for a framework to interpret their quality. Manual assessments of data quality may still be possible for physically collected samples. Yet, automated data collection often necessitates the automation of data quality assessments as well, which can become difficult to interpret for large data sets. Thus, in order to add the final automation step for assessing data quality, we have presented an integrated, transferable, and scalable data quality framework methodology. These methods could be readily refined and expanded upon to develop more complex summarization methodologies for specific issues and/or data types.

The data quality framework is flexible and allows its components to be altered or recombined in various ways to support specific areas of interest. For instance, the framework supports assessing the track record of a sensor because all of the sensor test and quality analysis information remains embedded. Thus, a sensor's track record is represented through the quality report and quality summary. The time period that is summarized through the data quality framework could be altered if one wanted to examine a sensor's track record at different granularities.

Similarly, a degrading sensor could be identified by assessing its quality metrics for a particular test of interest. There are a number of quality analyses that have and/or could be developed to aid in tracking the degradation of a sensor, and the quality framework could be employed to summarize the results so that they could be readily interpreted. Additionally, quality metrics could be incorporated into automated routines to generate trouble tickets for specific issues, and could be used as inputs for further in-depth quality analyses.

The most notable property of the presented data quality framework is its modular nature, which permits its use with a freely selectable array of data types, tests, and processing steps. The quality framework can be constructed or deconstructed in different forms, in order to allow it to be used on different spatial and temporal scales. For example, terrestrial physical sampling such as species counts, may initially only utilize the 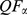 portion of the framework to determine whether or not an observation had 1 or more failed quality analyses. Likewise, physical samples collected for aquatic analyses, such as phytoplankton biomass, may employ similar components of the quality framework. On the other hand, aquatic sensor measurements are likely to use components of the quality framework that are similar to those used by the TRAAT sensor. The framework can also be arranged in a cascading manor to handle multiple data streams and processing steps that result in a single estimate, e.g., of the surface-atmosphere exchange.

## Supporting Information

Data S1 **The triple redundant air aspirated temperature data used in the manuscript are provided in the zip file “Data S1”.** The zip files includes two directories: Directory “Final Modified Data” contains the calibrated error-injected data and directory “Raw Data” provides the uncalibrated raw sensor measurements from the three PRTs together with their calibration equations and sensor specific calibration coefficients.
(ZIP)
